# Cratenin, a Rare Oxylipin Marking Kleptopredation in Aeolid Nudibranchs

**DOI:** 10.1021/acs.jnatprod.5c01409

**Published:** 2026-01-03

**Authors:** Giulia Quaini, Federica Albiani, Marcello Ziaco, Laura Fioretto, Olimpia Follero, Carmela Gallo, Giuliana d’Ippolito, Emiliano Manzo, Genoveffa Nuzzo, Angelo Fontana

**Affiliations:** † Institute of Biomolecular Chemistry, Consiglio Nazionale delle Ricerche, Via Campi Flegrei 34, 80078 Pozzuoli, Italy; ‡ Department of Biology, University of Naples “Federico II”, Via Cupa Nuova Cinthia 21, 80126 Napoli, Italy; § Institute of Biomolecular Chemistry, Consiglio Nazionale delle Ricerche, Via Paolo Gaifami, 18, 95126 Catania, Italy

## Abstract

Marine mollusks of the order Nudibranchia produce a wide array of secondary metabolites that play key roles in predator–prey interactions and often exhibit remarkable bioactivity. In this study, a chemical investigation of the Mediterranean aeolid nudibranchs *Cratena peregrina* and *Paraflabellina ischitana* led to the isolation and characterization of a novel oxylipin, designated cratenin (**1**). This unique metabolite shows an unusual alkylated monosubstituted tetrahydrofuran (THF) moiety, a structural feature rarely encountered among marine natural products. The planar structure of **1** was fully elucidated by high-resolution mass spectrometry (HRMS) and comprehensive 1D and 2D NMR spectroscopy, while the absolute configuration of the substituted THF ring was rigorously established by chemical degradation, derivatization, and NMR-based stereochemical comparison with known diasteromeric derivatives of (tetrahydrofuran-2-yl)­methanol. The co-occurrence of cratenin (**1**) in the hydrozoan *Eudendrium racemosum*, a known prey of *C. peregrina* and other aeolid nudibranchs, strongly suggests its role as a semiochemical which mediates predator–prey interactions. The proposed biosynthetic origin of cratenin (**1**) from algal docosahexaenoic acid (DHA) further corroborates the hypothesis of dietary acquisition and provides compelling molecular evidence for kleptopredation, a sophisticated foraging behavior where nudibranchs consume prey that has recently ingested phytoplankton. In this view, this study reveals a clear metabolic and ecological link connecting phytoplankton, hydrozoans, and nudibranchs, underscoring the pivotal role of lipid-derived natural products in shaping chemical communication and interphyletic trophic interactions involving opisthobranchs.

## Graphical abstract

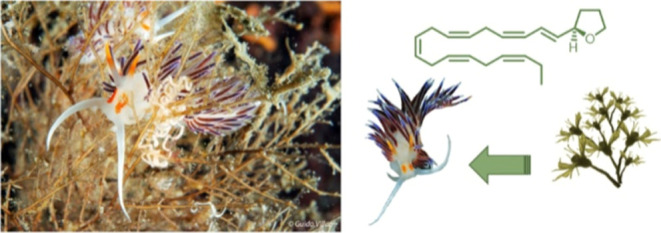

Marine Heterobranchia are exemplary models in chemical ecology due to their intricate trophic and chemical interactions with other organisms within their ecological niches.^1–4^ These soft-bodied gastropods have evolved sophisticated strategies for chemical defense, relying on a broad spectrum of secondary metabolites with deterrent, toxic, or signaling functions.^5^ The origins of these compounds are diverse, with several being directly sequestered from prey, some undergoing enzymatic transformation or synthesis within the host.^6^ This metabolic versatility has made heterobranch mollusks a prolific source of structurally unique natural products with significant ecological and pharmacological relevance.^7–9^

Among heterobranchs, aeolid nudibranchs have attracted sustained interest for their ability to co-opt functional traits from their cnidarian prey, including the storage of nematocysts within specialized structures called cnidosacs.^10^ Beyond their physical defense mechanisms, many of these species also accumulate prey-derived metabolites, which are retained to serve in chemical defense. These adaptations underline a complex trophic network where feeding behaviors are tightly linked to the acquisition of bioactive molecules.

Considerable progress has been made in elucidating the structural diversity and origin of secondary metabolites in marine heterobranchs.^7–9,11^ Accumulating evidence, from our work and those of others, indicates that dietary and *de novo* biosynthesis play a central role in shaping their chemical repertoires composed of polyketides, terpenoids, and lipid-derived structures with notable bioactivities and ecological functions.^12^ In addition, strong correlation between dietary specialization and distinct chemical profiles have been documented, supporting scenarios of trophic metabolite transfer and chemical adaptation in response to prey availability.

Within this context, we recently focused on *Cratena peregrina* and *Paraflabellina ischitana*, two visually distinctive aeolid nudibranchs of the Facelinidae family endemic in the Mediterranean Sea. These species are characterized by a translucent body and vividly pigmented cerata implicated not only in respiration and digestion but also in effective defense mechanisms.^13^ *C. peregrina* primarily preys upon hydroids of the genus *Eudendrium*, particularly*Eudendrium racemosum*.^14^ In addition to incorporating cnidoblasts for physical deterrence,*C. peregrina*exhibits dietary preferences that reflect complex feeding strategies. Recent ecological observations suggest that*C. peregrina*engages in kleptopredation, an opportunistic feeding behavior where the nudibranch preferentially consumes hydrozoans that have recently ingested phytoplankton.^15^ This behavior results in indirect planktivory, potentially increasing the yield of nutritionally beneficial or chemically enriched metabolites.

Here, we report an investigation of the chemical ecological relationship of *C. peregrina*and *P. ischitana* with their prey *E. racemosum*, which led to the isolation of cratenin (**1**), a novel secondary metabolite belonging to the oxylipin family.

Given the well-established involvement of oxylipins as mediators in marine environments,^16^ this study focused on the comprehensive chemical characterization of **1**, including the unambiguous determination of its absolute configuration and its possible biosynthetic origin from docosahexaenoic acid (DHA), a key ω-3 polyunsaturated fatty acid commonly produced by marine microalgae. In addition, we explored the molecular basis of the proposed kleptopredation strategy within the predator–prey relationship between the mollusk and the cnidarian. The detection of compound **1** in two genera of aeolid nudibranchs suggests its potentially utility as a chemical marker of specific dietary inputs within this group of mollusks, providing new insights into the metabolic pathways underlying trophic interactions in marine systems.
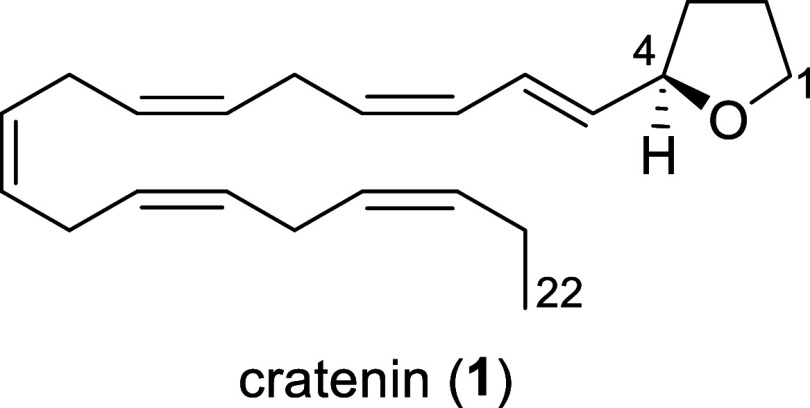


## Results and Discussion

*C. peregrina* and *P. ischitana* nudibranchs were subjected to acetone extraction by first gentle sonication and then grinding the whole organisms to yield mantle-enriched and total organic extracts, respectively. These fractions, along with the acetone extract of the hydrozoan *E. racemosum*, were analyzed by thin layer chromatography (TLC). A distinct and diagnostic UV–visible spot (*R*_*f*_ 0.8 in petroleum ether/ethyl ether 6:4, v/v) was consistently observed across all samples, with marked enrichment in the mantle of the aeolid nudibranchs. Subsequent purification of *C. peregrina* and *P. ischitana* extracts by silica chromatography led to the isolation of the new metabolite cratenin (**1**).

High-resolution electrospray ionization mass spectrometry (HRESIMS) of compound **1** (1 mg) revealed a sodium adduct ion [M + Na]^+^ at *m*/*z* 335.2342, consistent with the molecular formula C_22_H_32_O and seven degrees of unsaturation (Figure S12). The ^1^H NMR spectrum (600 MHz, C_6_D_6_) displayed multiple olefinic resonances between δ_H_ 5.3 and 6.4 ppm, alongside signals at δ_H_ 4.25 (multiplet), 3.77 (ddd, *J* = 15.0, 7.0, 1.7 Hz), and 3.60 ppm (ddd, *J* = 15.0, 7.8, 1.5 Hz) attributable to protons on oxygen-bearing methine and methylene groups, respectively. Additional high-field resonances were consistent with an unfunctionalized linear alkyl chain (Table 1, Figure S1).

**Table 1:** NMR Spectroscopic Data (600 MHz) for Cratenin **1** in (a) C_6_D_6_ and (b) CDCl_3_

	(a)		(b)	
position	δ_C_, type	δ_H,_ m (*J* in Hz)	δ_C_, type	δ_H,_ m (*J* in Hz)
1	67.6, CH_2_	3.77, ddd (15.0, 7.0, 1.7)	68.1, CH_2_	3.90, ddd (15.0, 7.0, 1.7)
		3.60, ddd (15.0, 7.8, 1.5)		3.77, ddd (15.0, 7.8, 1.5)
2	25.6, CH_2_	1.56, m	25.9, CH_2_	1.90, m
		1.49, m		1.93, m
3	31.9, CH_2_	1.68, m	32.5, CH_2_	2.02, m
		1.35, m		1.63, m
4	79.1, CH	4.25, m	79.5, CH	4.30, m
5	130.4, CH	5.63, m	131.8, CH	5.58, m
6	131.0, CH	6.31, m	131.1, CH	6.18, m
7	130.7, CH	6.06, m	130.2, CH	6.03, m
8	132.3, CH	5.58, m	134.2, CH	5.67, m
9	32.7, CH_2_	2.00, m	32.6, CH_2_	2.16, m
10	32.7, CH_2_	2.00, m	32.6, CH_2_	2.16, m
11	131.0, CH	5.61, m	131.8, CH	5.54, m
12	130.0, CH	6.29, m	131.1, CH	6.19, m
13	130.7, CH	6.05, m	130.2, CH	6.05, m
14	132.4, CH	5.55, m	134.2, CH	5.65, m
15	35.5, CH_2_	2.67, m	35.6, CH_2_	2.78, m
16	131.1, CH	5.56, m	134.2, CH	5.65, m
17	128.0, CH	5.44, m	129.9, CH	5.37, m
18	30.3, CH_2_	2.72, m	25.6, CH_2_	2.83, m
19	131.3, CH	5.45, m	129.9, CH	5.37, m
20	129.0, CH	5.44, m	131.1, CH	5.41, m
21	25.9, CH_2_	1.96, m	26.1, CH_2_	1.98, m
22	13.8, CH_3_	0.94, t (7.0)	14.4, CH_3_	0.96, t (7.0)

COSY NMR experiments defined a contiguous spin system extending from C1 to C7 of **1**. HMBC correlations in C_6_D_6_between methylene protons at δ_H_ 3.77–3.60 and the carbon resonance at δ_C_ 79.1 (methine C-4) suggested the presence of a monosubstituted tetrahydrofuran (THF) ring (Figure S5). The remaining six degrees of unsaturation were assigned to multiple double bonds distributed along the aliphatic chain. The complexity and multiplicity of olefinic signals indicated the coexistence of geometric isomers, likely resulting from spontaneous *cis*–*trans* isomerization during sample handling. Nevertheless, this did not prevent the assignment of the absolute configuration at C-4 by chemical transformation of the natural product (Figure 1). To this aim, nearly 1 mg of compound **1** was subjected to ozonolysis and *in situ* reduction by DIBAL. The resulting alcohol was esterified by (*R*)-naproxen to yield sufficient quantity of compound **1a** for ^1^H NMR analysis. In parallel, enantiomerically pure (*R*)- and (*S*)-(tetrahydrofuran-2-yl)­methanol standards were derivatized with (*R*)-naproxen to afford, respectively, the NMR-distinguishable diastereomeric esters **2** and **3** showing opposite configuration at the chiral carbon of the tetrahydrofuran ring (Figures S14 and S15). Comparison of the ^1^H NMR spectra of these reference compounds with that of the natural product derivative revealed a clear correspondence between derivative **1a** and compound **2**, thus establishing the *R* absolute configuration at C-4 of cratenin (**1**).

**Figure 1:**
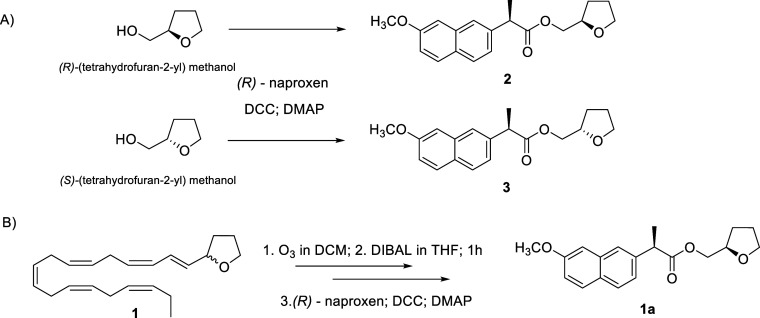
(A) Derivatization of pure enantiomers to achieve the diastereomers **2** and **3** by esterification with (*R*)-naproxen; (B) Flow of reactions to define absolute stereochemistry of cratenin **1**.

The chirality of C4 proved the enzymatic origin of **1**, thus indicating a defined biosynthetic origin for this novel lipid. The monosubstituted tetrahydrofuran (THF) ring found in cratenin (**1**) represents a relatively rare, yet documented, structural motif among bioactive marine oxylipins.^17,18^ In nature, the biosynthesis of such five-membered ether rings from polyunsaturated fatty acids (PUFAs) typically proceeds via a mechanism involving stereoselective enzymatic oxidation (often catalyzed by lipoxygenases), followed by a chemoselective cyclization event. Specifically, we hypothesize that the C22 precursor docosahexaenoic acid (DHA), an ω-3 PUFA abundant in marine microalgae, undergoes a sequence of oxidations and intramolecular cyclizations to yield **1** via a lipoxygenase (LOX)-mediated pathway.^19^ In particular, in line with previous investigations on LOX activity on eicosanoid acids in diatoms,^20–24^ the plausible multistep process is suggested to start from peroxidation at C-4 of DHA catalyzed by a 4*R*-LOX (Figure 2). Subsequent reduction of the peroxide function allows a spontaneous cyclization that would then generate a five-member oxygenated intermediate, which can be converted to cratenin (**1**) through additional reductive steps. Notably, throughout this process, the final product retains the stereochemistry at C-4 introduced by the regio- and stereoselectivity characteristic of the initial enzymatic lipoxygenation.^24^

**Figure 2:**
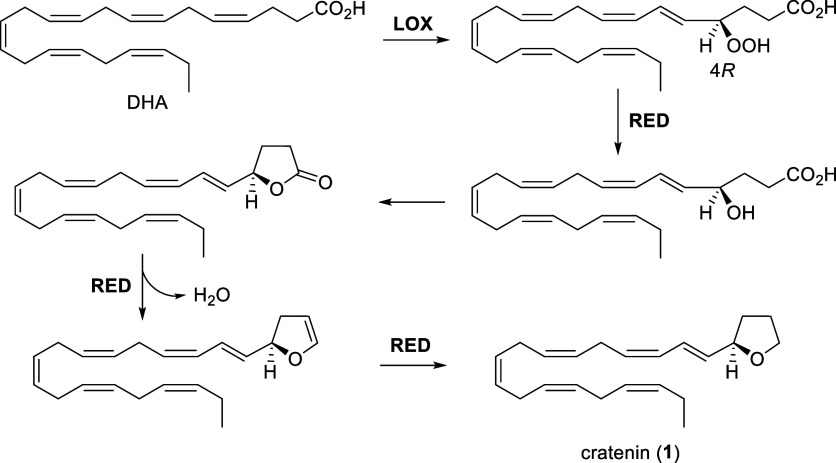
Proposal of the biosynthesis of cratenin (**1**). LOX: lipoxygenase; RED: reductase.

This proposed biosynthetic pathway for cratenin (**1**) is analogous to established routes documented for the pheromones petromyroxols and petromyric acids A and B, a family of ether lipids isolated from larval sea lamprey *Petromyzon marinus* L.^25^ Other marine THF-containing fatty acid derivatives in the marine environment are mutafurans A–G isolated from the sponge *Xextospongia testudinaria*, and aspericacids A and B from the fungus associated with the sponge *Haliclona* sp.^26,27^ Among terrestrial organisms, a notable example is the pollinator attractant 2-(tetrahydrofuran-2-yl)-acetic acid reported in the sexually deceptive orchid *Cryptostylis ovata*.^28^

These parallel reports in the mollusks and the cnidarian strengthen the hypothesis that such a metabolic pathway is highly conserved across various ecosystems and supports the conclusion that cratenin (**1**) is a natural product acquired through trophic transfer from *Eudendrium* hydrozoans,^29–31^ such as *E. racemosum*, following the initial biotransformation of microalgal fatty acids. In addition, the proposed biosynthetic pathway from DHA not only substantiates the structural characterization of the novel oxylipin but also helps to establish the precise position and configuration of the double bonds as depicted in Figure 2.

Beyond the incorporation of nematocysts for physical deterrence, it has been reported that *C. peregrina* exhibits several biochemical and structural adaptations that enhance its interaction with toxic prey. These include the presence of intracellular chitin granules in the epidermis and digestive tract and the release of protective spindle-like structures in response to nematocyst discharge.^32,33^ Collectively, these features suggest a complex defensive strategy combining behavioral, structural and visual (i.e., aposematic coloration) mechanisms of interaction. In this framework, the presence of a previously unreported oxylipin-like metabolite in *C. peregrina* may represent an additional component of its adaptive toolkit. While further ecological and functional studies are required to clarify the precise role of cratenin (**1**), the incorporation and bioaccumulation of this lipophilic metabolite could reflect a broader biochemical strategy to exploit cnidarian defenses not only mechanically but also chemically.

Willis et al.^15^ first described kleptopredation in *C. peregrina*, wherein the nudibranch exploits hydroids that have recently captured phytoplankton, thereby incorporating planktonic resources into its diet. This subsidized predation has been considered advantageous for exploiting ephemeral food sources. We suggest that cratenin (**1**), as a new oxylipin biosynthetically related to a fatty acid such as DHA particularly abundant in diatoms, provides the first metabolic validation of this ecological interaction and highlights a metabolic link bridging phytoplankton, hydrozoans and nudibranchs. It is worth noting that the phytoplankton community associated with *E. racemosum* is reported as predominantly composed of diverse diatoms.^35^ Consistent with our hypothesis, recent studies have reported similar trophic association in other aeolidaceans, such as *Sakuraeolis marhe* feeding on the hydrozoan *Zanclea* sp.^34^

Oxylipins are known as semiochemicals in several natural contexts, including physiological and pathological processes. The biological role of these compounds has been especially studied in mammalians and higher plants, although a varied and very high concentration of these products have also been reported from marine diatoms, a major class of planktonic microalgae. Oxylipins, isolated from diatoms, are reported to induce teratogenic effects in copepods.^16c,16d^ However, despite the negative impact observed in marine invertebrates, some volatile oxylipins were also proposed as odor attractant compounds, thus suggesting that the function of oxylipins can be very complex and could be strongly dependent on the ecological context of the chemical interactions. Given the established functions of oxylipins and related lipid mediators in signaling, wound response and interspecies communication, it is reasonable to propose that cratenin, with its lipophilicity and structural features, may play an analogous ecological role as a signaling molecule in chemical defense, intraspecific communication or by contributing to kleptopredation by modulating predator–prey interactions. These possibilities warrant further dedicated bioassays and ecological evaluations.

In conclusion, cratenin (**1**) emerges as a valuable chemical marker for understanding intricate trophic interactions and metabolic exchanges between aeolid nudibranchs, such as *C. peregrina* and *P. ischitana*, and *Eudendrium* hydrozoans, like *E. racemosum*. The finding expands the known chemical diversity of marine natural products in marine heterobranchs mollusks, and highlights their distinctive ecological ability to sequester and exploit bioactive molecules acquired directly from dietary sources. Notably, cratenin (**1**) can be regarded as a molecular signature of a cross-kingdom chemical interplay, suggesting a potential evolutionary dependency underlying these adaptations.

## Experimental Section

### General Experimental Procedures

Optical rotations were measured with a Jasco P-2000 digital polarimeter. UV spectra were recorded using a Jasco V-650 UV–vis spectrophotometer. NMR spectra were recorded on a Bruker DRX-600 spectrometer operating at 600 MHz for ^1^H and 150 MHz for ^13^C, equipped with a triple resonance inverse (TCI) CryoProbe. Chemical shifts (δ) are referenced to residual solvent signals: CDCl_3_ (δ_H_ 7.26, δ_C_ 77.0) and C_6_D_6_ (δ_H_ 7.16, δ_C_ 128.0). High-resolution electrospray ionization mass spectra (HRESIMS) were acquired using a Q-Exactive Hybrid Quadrupole-Orbitrap Mass Spectrometer (Thermo Scientific). TLC plates (silica gel 60 F254), silica gel powder (silica gel 60 0.063–0.200 mm), solvent and chemical reagent were from Merck (Darmstadt, Germany). Pure enantiomers (*R*)- and (*S*)-(tetrahydrofuran-2-yl)­methanol were purchased from Alina AA BLOCKS of San Diego (CA).

### Biological Material

Specimens of *C. peregrina* and *P. ischitana* were collected by snorkeling in May 2024 from hydranths of *E. racemosum* in Lake Miseno, Naples, Italy (40°47′31.1″N, 14°04′21.17″E). Fresh material was immediately processed for extraction. Voucher specimens are preserved at the Institute of Biomolecular Chemistry, National Research Council of Italy (ICB-CNR), Pozzuoli, Italy, under frozen conditions.

### Extraction and Isolation of *C. peregrina*

Fourteen individuals of *C. peregrina* were subjected to two successive acetone washes to enrich secondary metabolites localized in the mantle (mantle extract, 4.2 mg). The same specimens were subsequently homogenized and exhaustively extracted with acetone by sonication (3 × 5 mL) to yield a total crude extract (5.6 mg). Both extracts were concentrated under reduced pressure, combined and subjected to silica gel column chromatography using a gradient of petroleum ether (PE) and Et_2_O as eluents. Five fractions (A–E) were collected based on TLC profiles. Fraction C, eluted with PE/Et_2_O 95:5, displayed a UV-active spot and was further analyzed by NMR spectroscopy (Figures S1–S11) and HRESIMS (Figure S12), resulting in the isolation of compound **1** (cratenin, 0.2 mg).

### Extraction and Isolation of *P. ischitana* and *E. racemosum*

A single individual of *P. ischitana* was homogenized and extracted by acetone (3 × 5 mL). The resulting crude extract (3.9 mg) was fractionated by silica gel column chromatography using a PE/Et_2_O gradient, as described above for *C. peregrina*. This procedure afforded compound **1** (cratenin, 0.1 mg), which was identified by ^1^H NMR and HRESIMS.

Freshly collected *E. racemosum* was extracted (150 mg) by acetone and fractionated as described above to afford 1 mg of cratenin (**1**). The product was identified by ^1^H NMR and HRESIMS.

### Derivatization of Compound **1**

A solution of compound **1** (1 mg) in dry CH_2_Cl_2_ was cooled to −78 °C and subjected to ozonolysis by bubbling ozone (O_3_). After removal of excess ozone, the reaction mixture was treated with an excess of DIBAL (1 M in THF, 100 μL) and stirred at room temperature for 1 h. Due to the volatility of the intermediate alcohol, naproxen acid (5 mg), DCC (5 mg) and DMAP (2 mg) were added directly to the crude reaction mixture, which was then stirred overnight at room temperature. The solvent was gently evaporated under a nitrogen stream, and the residue was purified by silica gel pipet column chromatography using a PE/Et_2_O gradient to afford derivative **1a** (0.1 mg).

### Cratenin (**1**)

Colorless oil; [α]_D_^25^ −12° (*c* 0.006, CH_2_Cl_2_); UV (CH_2_Cl_2_) λ_max_ (log ε) 240 nm (3.69), 274 nm (3.39); ^1^H and ^13^C NMR data in C_6_D_6_ and CDCl_3_ are reported in Table 1; HRESIMS [M + Na]^+^ *m*/*z* 335.2342 (calcd for C_22_H_32_NaO_2_^+^ *m*/*z* 335.2345).

### Derivative **1a** (Naproxen Ester)

Diagnostic ^1^H NMR data (600 MHz, CDCl_3_): δ_H_ 7.70 (1H, d, *J* = 8.3 Hz), 7.69 (1H, *d*, *J* = 8.3 Hz), 7.64 (1H, br s), 7.38 (1H, br d, *J* = 8.0 Hz), 7.14 (1H, br d, *J* = 8.0 Hz), 7.10 (1H, br s), 4.05 (1H, m), 4.08–4.10 (2H, m), 3.91 (3H, s), 3.63 (1H, m), 3.62–3.58 (2H, m), 1.93–1.55 (2H, m), 1.70 (2H, m), 1.54 (3H, overlapped); HR-ESIMS [M + Na]^+^ *m*/*z* 337.1408 (calcd for C_19_H_22_NaO_4_^+^ *m*/*z* 337.1410).

## Supplementary Material



## Data Availability

NMR raw data are available and accessible at the following repository: https://cloud.icb.cnr.it/s/HReCn4TNRPaxQbj.

## Notes

^1^H NMR spectra of compounds **1**–**3** and derivative **1a**; 2D NMR spectra of compound **1** (PDF)

## References

[1] Avila C., Angulo-Preckler C. (2020). Bioactive Compounds from Marine Heterobranchs. Mar. Drugs.

[2] Dean L. J., Prinsep M. R. (2017). The Chemistry and
Chemical Ecology of Nudibranchs. Nat. Prod.
Rep..

[3] Bornancin L., Bonnard I., Mills S. C., Banaigs B. (2017). Chemical mediation
as a structuring element in marine gastropod predator-prey interactions. Nat. Prod. Rep..

[4] Winters A. E., Chan W., White A. M., van den Berg C. P., Garson M. J., Cheney K. L. (2022). Weapons or Deterrents? Nudibranch
Molluscs Use Distinct Ecological Modes of Chemical Defence against
Predators. J. Anim. Ecol..

[5] Cimino, G. ; Ghiselin, M. T. Chemical Defense and the Evolution of Opisthobranch Gastropods, Proceedings of the California Academy of Sciences; California Academy of Sciences, 2009: Vol. 60, ISBN: 0940228793, 9780940228795.

[6] Fontana A. (2006). Biogenetic
Proposals and Biosynthetic Studies on Secondary Metabolites of Opisthobranch
Molluscs. Prog. Mol. Subcell. Biol..

[7] Carroll A. R., Copp B. R., Grkovic T., Keyzers R. A., Prinsep M. R. (2025). Marine
natural products. Nat. Prod. Rep..

[8] Cimino, G. ; Ciavatta, M. L. ; Fontana, A. ; Gavagnin, M. Metabolites of Marine Opisthobranchs: Chemistry and Biological Activity. Bioactive Compounds from Natural Sources; Taylor & Francis; London, 2001; pp 577–637.

[9] Chen Z. H., Guo Y. W., Li X. W. (2023). Recent Advances on Marine Mollusk-Derived
Natural Products: Chemistry, Chemical Ecology and Therapeutical Potential. Nat. Prod. Rep..

[10] Greenwood P. G., Mariscal R. N. (1984). The Utilization of Cnidarian Nematocysts by Aeolid
Nudibranchs: Nematocyst Maintenance and Release in *Spurilla*. Tissue Cell.

[11] Moreiras-Figueruelo A., Nuzzo G., Galasso C., Sansone C., Crocetta F., Mazzella V., Gallo C., Barra G., Sardo A., Iuliano A., Manzo E., d’Ippolito G., Albrigtsen M., Andersen J. H., Ianora A., Fontana A. (2021). Probing the
Therapeutic Potential of Marine Phyla by SPE Extraction. Mar. Drugs.

[12] Bogdanov A., Hertzer C., Kehraus S., Nietzer S., Rohde S., Schupp P. J., Wägele H., König G. M. (2017). Secondary Metabolome and Its Defensive Role in the
Aeolidoidean *Phyllodesmium longicirrum*, (Gastropoda,
Heterobranchia, Nudibranchia). Beilstein J.
Org. Chem..

[13] Aguado F., Marin A. (2007). Warning Coloration
Associated with Nematocyst-Based Defences in Aeolidiodean
Nudibranchs. J. Mollus. Stud..

[14] Di
Camillo C. G., Betti F., Bo M., Martinelli M., Puce S., Vasapollo C., Bavestrello G. (2012). Population
Dynamics of *Eudendrium racemosum* (Cnidaria, Hydrozoa)
from the North Adriatic Sea. Mar. Biol..

[15] Willis T. J., Berglöf K. T. L., McGill R. A. R., Musco L., Piraino S., Rumsey C. M., Fernández T. V., Badalamenti F. (2017). Kleptopredation:
A Mechanism to Facilitate Planktivory in a Benthic Mollusc. Biol. Lett..

[16] Deng Y., Vallet M., Pohnert G. (2022). Temporal and
Spatial Signaling Mediating the Balance of the Plankton Microbiome. Annu. Rev. Mar. Sci..

[17] Fernández-peña L., Díez-poza C., González-andrés P., Barbero A. (2022). The Tetrahydrofuran Motif in Polyketide Marine Drugs. Mar. Drugs.

[18] González-Andrés P., Fernández-Peña L., Díez-Poza C., Barbero A. (2022). The Tetrahydrofuran Motif in Marine Lipids and Terpenes. Mar. Drugs.

[19] Nanjappa D., D’Ippolito G., Gallo C., Zingone A., Fontana A. (2014). Oxylipin Diversity
in the Diatom Family Leptocylindraceae Reveals DHA Derivatives in
Marine Diatoms. Mar. Drugs.

[20] Fontana A., D’Ippolito G., Cutignano A., Miralto A., Ianora A., Romano G., Cimino G. (2007). Chemistry of Oxylipin Pathways in
Marine Diatoms. Pure Appl. Chem..

[21] Jagusch H., Baumeister T. U. H., Pohnert G. (2020). Mammalian-Like Inflammatory and Pro-Resolving
Oxylipins in Marine Algae. Chembiochem..

[22] Adelfi M. G., Vitale R. M., d’Ippolito G., Nuzzo G., Gallo C., Amodeo P., Manzo E., Pagano D., Landi S., Picariello G., Ferrante M. I., Fontana A. (2019). Patatin-like Lipolytic
Acyl Hydrolases and Galactolipid Metabolism in Marine Diatoms of the
Genus *Pseudo-Nitzschia*. BBA-Mol.
Cell. Biol. Lipids..

[23] D’Ippolito G., Lamari N., Montresor M., Romano G., Cutignano A., Gerecht A., Cimino G., Fontana A. (2009). 15S-Lipoxygenase Metabolism
in the Marine Diatom *Pseudo-Nitzschia delicatissima*. New Phytol..

[24] Lamari N., Ruggiero M. V., d’Ippolito G., Kooistra W. H. C. F., Fontana A., Montresor M. (2013). Specificity
of Lipoxygenase Pathways
Supports Species Delineation in the Marine Diatom Genus *Pseudo-Nitzschia*. PLoS One.

[25] Li K., Huertas M., Brant C., Chung-Davidson Y. W., Bussy U., Hoye T. R., Li W. (2015). (+)- and (−)-Petromyroxols:
Antipodal Tetrahydrofurandiols from Larval Sea Lamprey (*Petromyzon
marinus* L.) That Elicit Enantioselective Olfactory Responses. Org. Lett..

[26] Zhou X., Lu Y., Lin X., Yang B., Yang X., Liu Y. (2011). Brominated
Aliphatic Hydrocarbons and Sterols from the Sponge *Xestospongia
testudinaria* with Their Bioactivities. Chem. Phys. Lipids.

[27] Liu Y., Ding L., Zhang Z., Yan X., He S. (2020). New Antifungal
Tetrahydrofuran Derivatives from a Marine Sponge-Associated Fungus *Aspergillus* sp. LS78. Fitoterapia.

[28] Bohman B., Weinstein A. M., Phillips R. D., Peakall R., Flematti G. R. (2019). 2-(Tetrahydrofuran-2-Yl)­Acetic
Acid and Ester Derivatives as Long-Range Pollinator Attractants in
the Sexually Deceptive Orchid *Cryptostylis ovata*. J. Nat. Prod..

[29] Martin R. (2003). Management
of Nematocysts in the Alimentary Tract and in Cnidosacs of the Aeolid
Nudibranch Gastropod *Cratena peregrina*. Mar. Biol..

[30] Lombardo A., Marletta G. (2020). New Data on the Seasonality of *Flabellina affinis* (Gmelin, 1791) and *Cratena peregrina* (Gmelin, 1791)
(Gastropoda Nudibranchia) in the Ionian Sea, Central Mediterranean. Biodivers. J..

[31] Martin R., Walther P. (2002). Effects of Discharging
Nematocysts When an Eolid Nudibranch
Feeds on a Hydroid. J. Mar. Biol. Assoc. U.K..

[32] Martin R., Hild S., Walther P., Ploss K., Boland W., Tomaschko K. H. (2007). Granular
Chitin in the Epidermis of Nudibranch Molluscs. Biol. Bull..

[33] Martin R., Tomaschko K. H., Walther P. (2007). Protective Skin Structures in Shell-Less
Marine Gastropods. Mar. Biol..

[34] Romagnoli T., Totti C., Accoroni S., De Stefano M., Pennesi C. (2014). SEM Analysis of the Epibenthic Diatoms
on *Eudendrium
racemosum* (Hydrozoa) from the Mediterranean Sea. Turk. J. Bot..

[35] Maggioni D., Furfaro G., Solca M., Seveso D., Galli P., Montano S. (2023). Being Safe, but Not Too Safe: A Nudibranch Feeding
on a Bryozoan-Associated Hydrozoan. Diversity.

