# Supplementary material for: Cratenin, a Rare Oxylipin Marking Kleptopredation in Aeolid Nudibranchs
Source: J Nat Prod. 2026 Jan 3;89(5):1407–13. doi: 10.1021/acs.jnatprod.5c01409 (PMC13200242; doi:10.1021/acs.jnatprod.5c01409)

Supporting information

Cratenin, a rare oxylipin marking kleptopredation in aeolid nudibranchs

Giulia Quaini[1,2], Federica Albiani[1,2], Marcello Ziaco[1,2], Laura Fioretto[1], Olimpia Follero[1], Carmela Gallo[1], Giuliana d'Ippolito[1], Emiliano Manzo[1], Genoveffa Nuzzo[1,] and Angelo Fontana[1,2,*].*

**genoveffa.nuzzo@cnr.it; angelo.fontana@cnr.it*

[1]Institute of Biomolecular Chemistry, Consiglio Nazionale delle Ricerche, Via Campi Flegrei 34, 80078 Pozzuoli, Italy;

[2]Department of Biology, University of Naples "Federico II", Via Cupa Nuova Cinthia 21, 80126 Napoli, Italy

S1. ^{1}H NMR (600 MHz, C_6D_6) spectrum of compound **1**

S2. COSY NMR (600 MHz, C_6D_6) spectrum of compound **1**

S3. TOCSY NMR (600 MHz, C_6D_6) spectrum of compound **1**

S4. HSQC NMR (600 MHz, C_6D_6) spectrum of compound **1**

S5. HMBC NMR (600 MHz, C_6D_6) spectrum of compound **1**

S6. ^{1}H NMR (600 MHz, $CDCl_3$) spectrum of compound **1**

S7. COSY NMR (600 MHz, $CDCl_3$) spectrum of compound **1**

S8. TOCSY NMR (600 MHz, $CDCl_3$) spectrum of compound **1**

S9. HSQC NMR (600 MHz, $CDCl_3$) spectrum of compound **1**

S10. HMBC NMR (600 MHz, $CDCl_3$) spectrum of compound **1**

S11. ^{13}C NMR (150 MHz, $CDCl_3$) spectrum of compound **1**

S12. HRESIMS of compound **1**

S13. UV of compound **1** (MeOH)

S14. ^{1}H NMR (600 MHz, $CDCl_3$) of compound **2**

S15. ^{1}H NMR (600 MHz, $CDCl_3$) of compound **3**

S16. Comparison between ^{1}H NMR (600 MHz, $CDCl_3$) spectra of compounds **2**, **3** and derivative of **1**

S1. ^{1}H NMR (600 MHz, C_6D_6) spectrum of compound **1.**

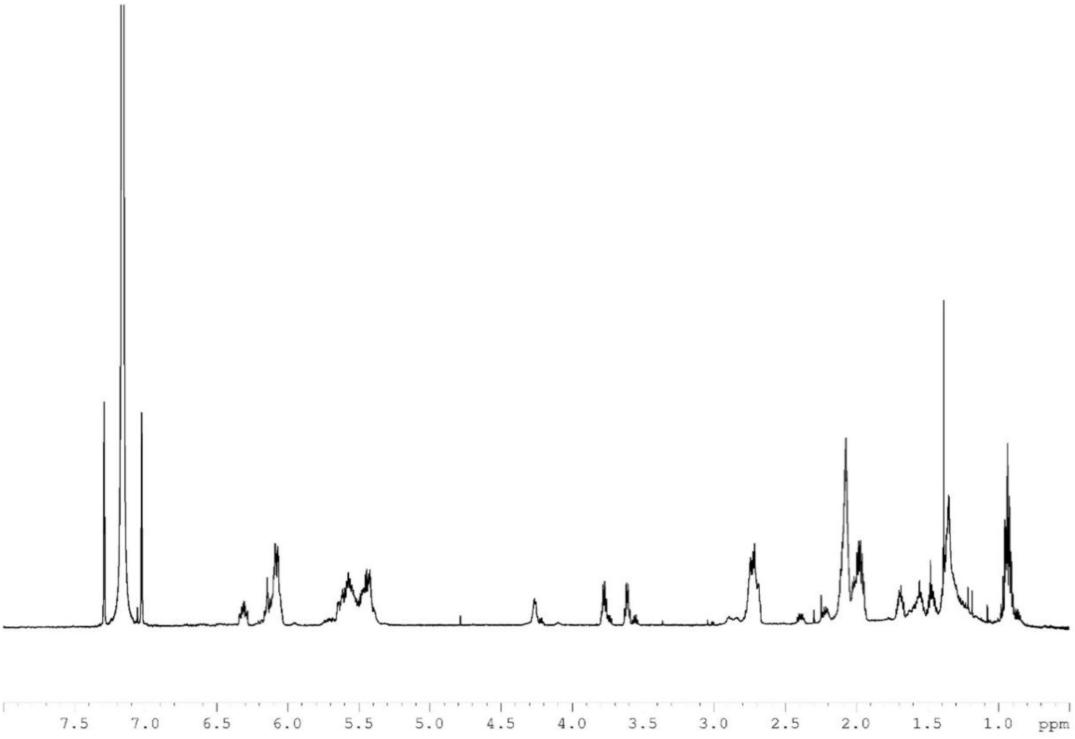

S2. COSY NMR (600 MHz, C_6D_6) of compound **1**.

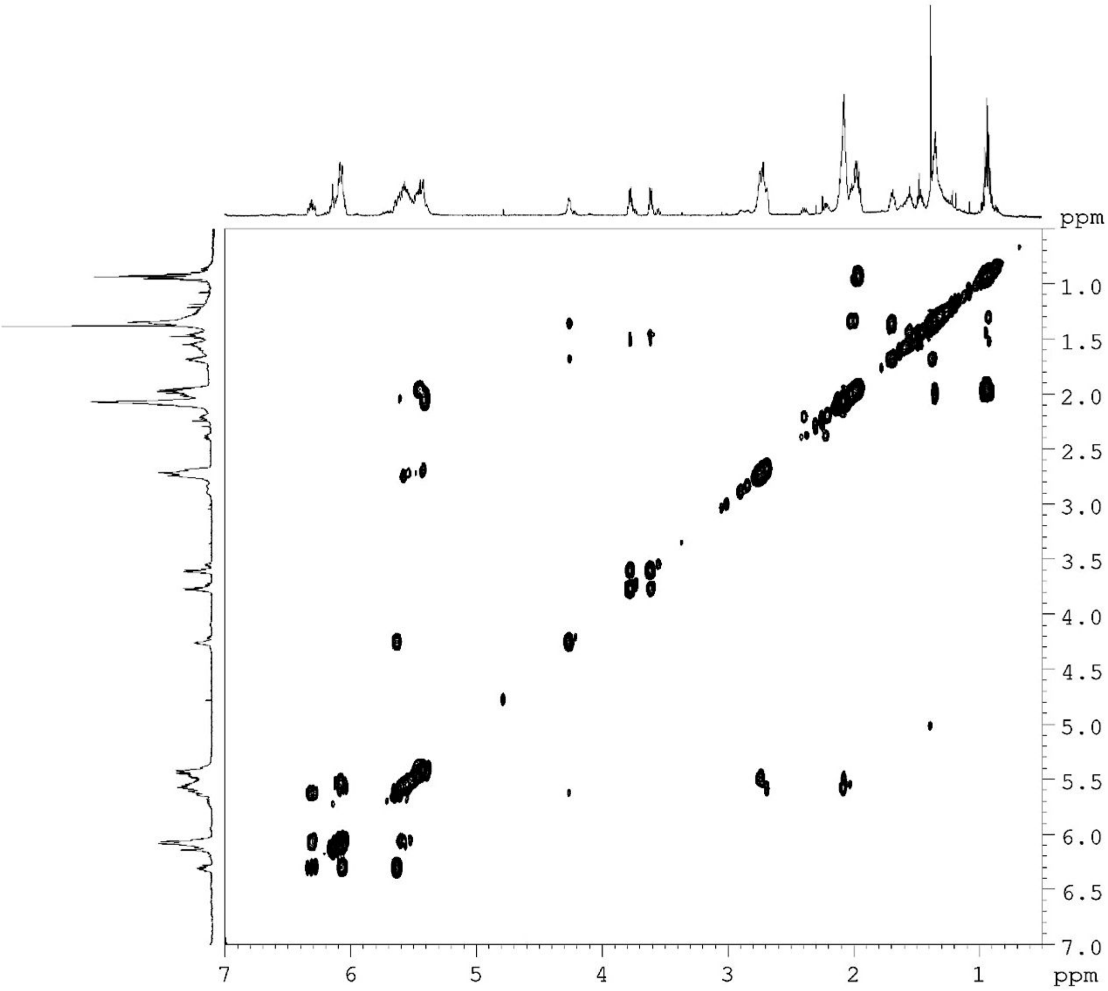
ppm
1.0
1.5
2.0
2.5
3.0
3.5
4.0
4.5
5.0
5.5
6.0
6.5
7.0
7
6
5
4
3
2
1
ppm

S3. TOCSY NMR (600 MHz, C_6D_6) of compound **1**.

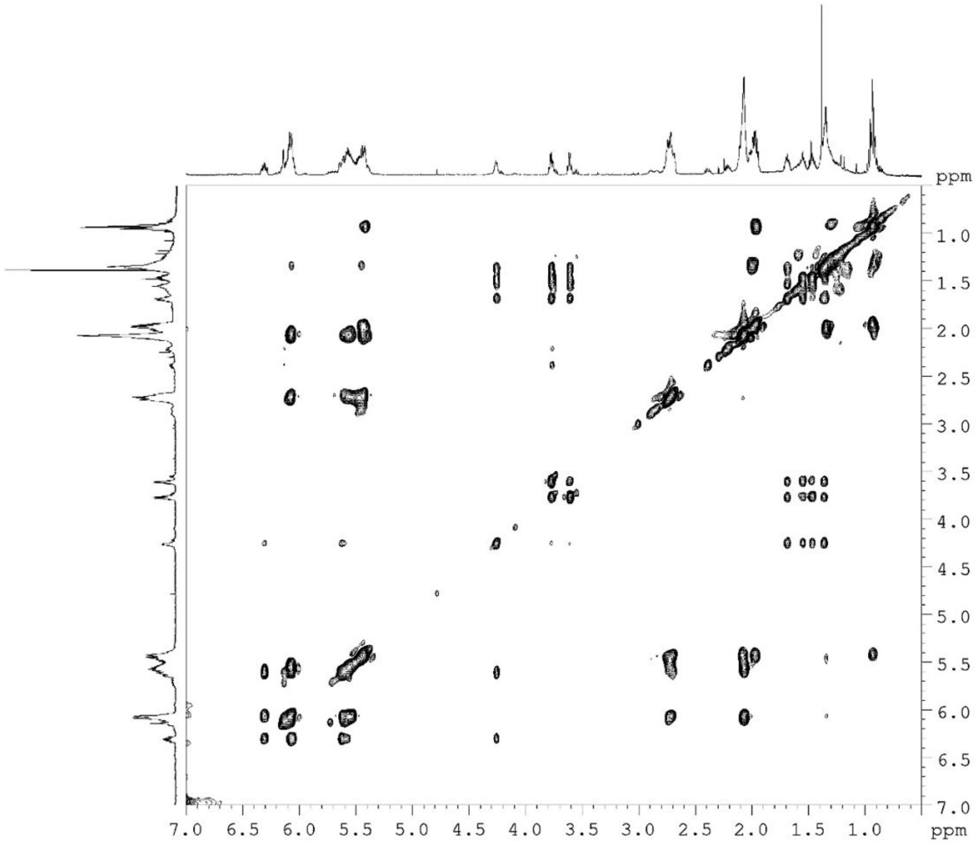

S4. HSQC NMR (600 MHz, C_6D_6) of compound **1**.

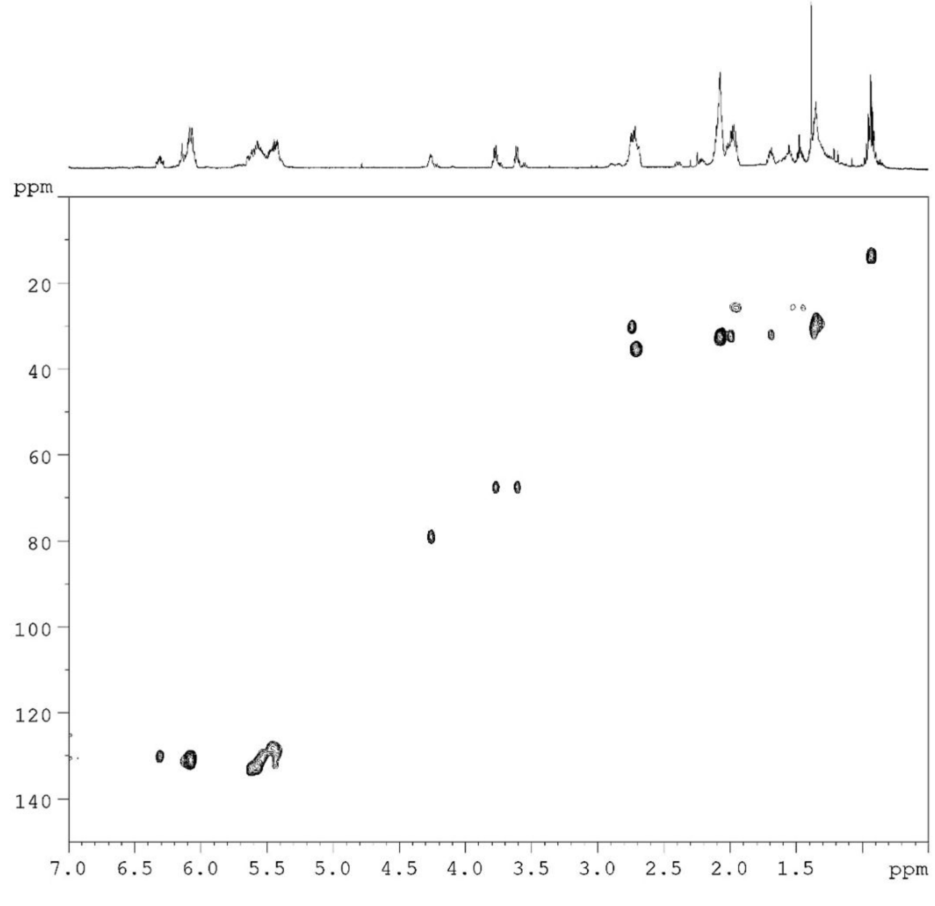

S5. HMBC NMR (600 MHz, C_6D_6) of compound **1**.

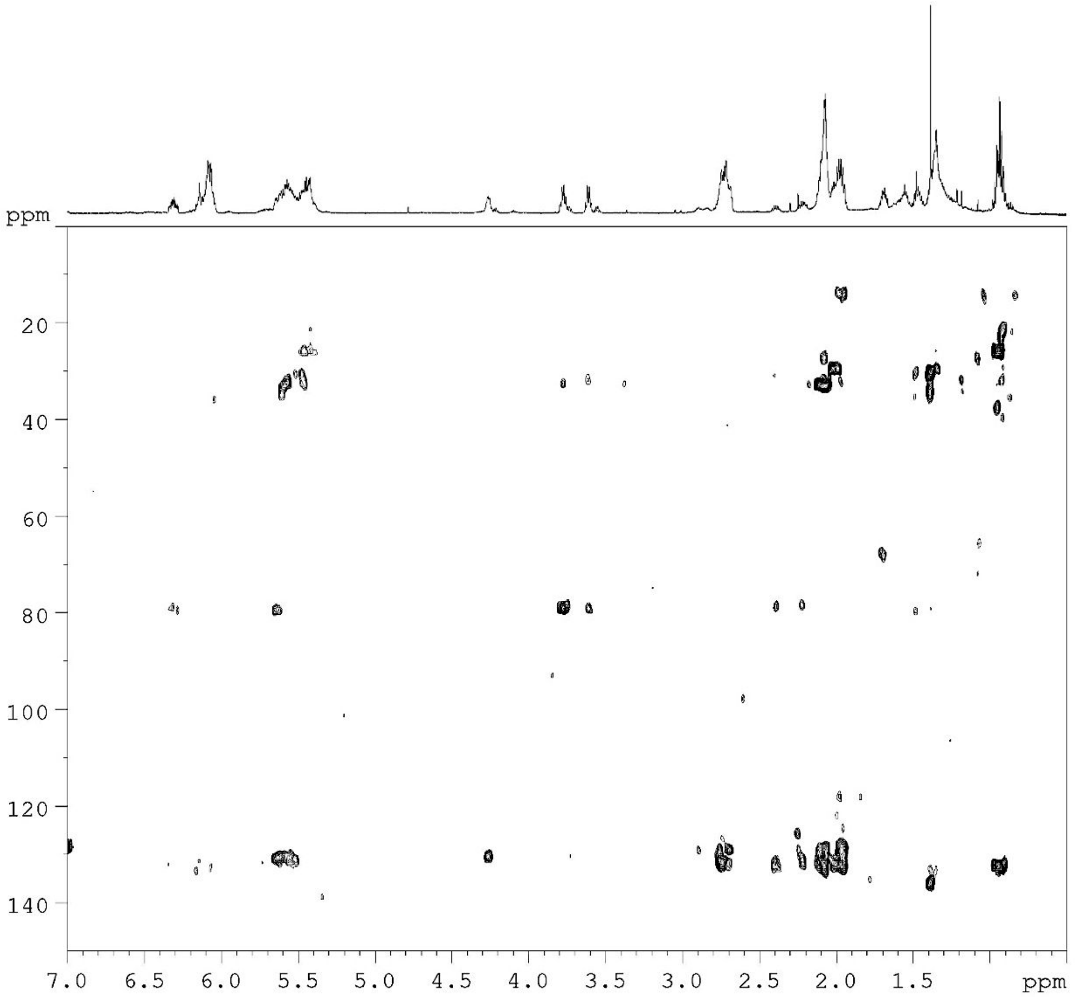

S6. ^1H NMR (600 MHz, $CDCl_3$) spectrum of compound **1**.

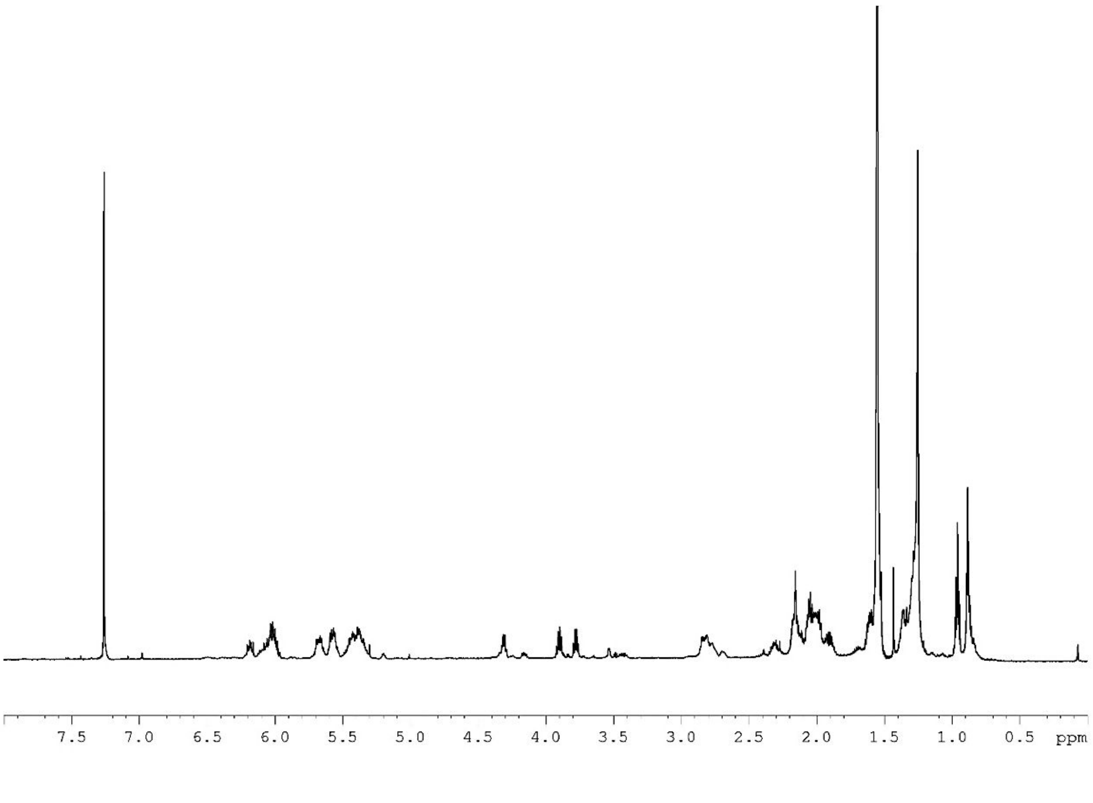

S7. COSY NMR (600 MHz, $CDCl_3$) of compound **1**.

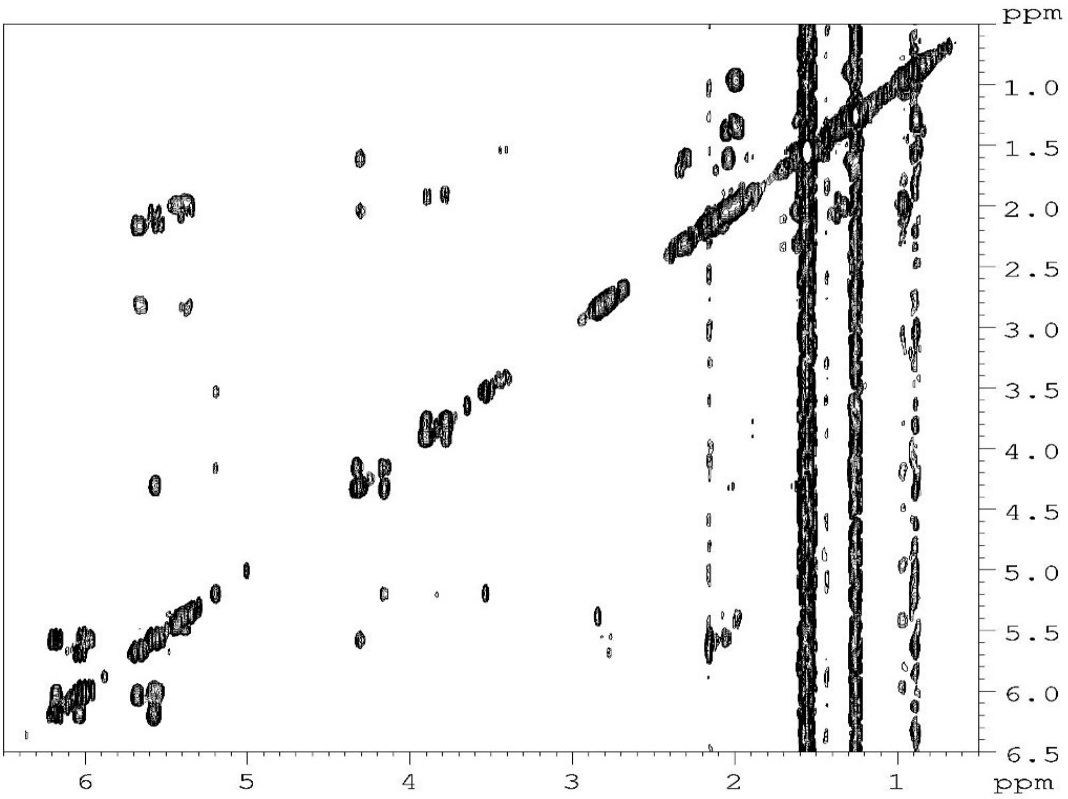

S8. TOCSY NMR (600 MHz, $CDCl_3$) of compound **1**.

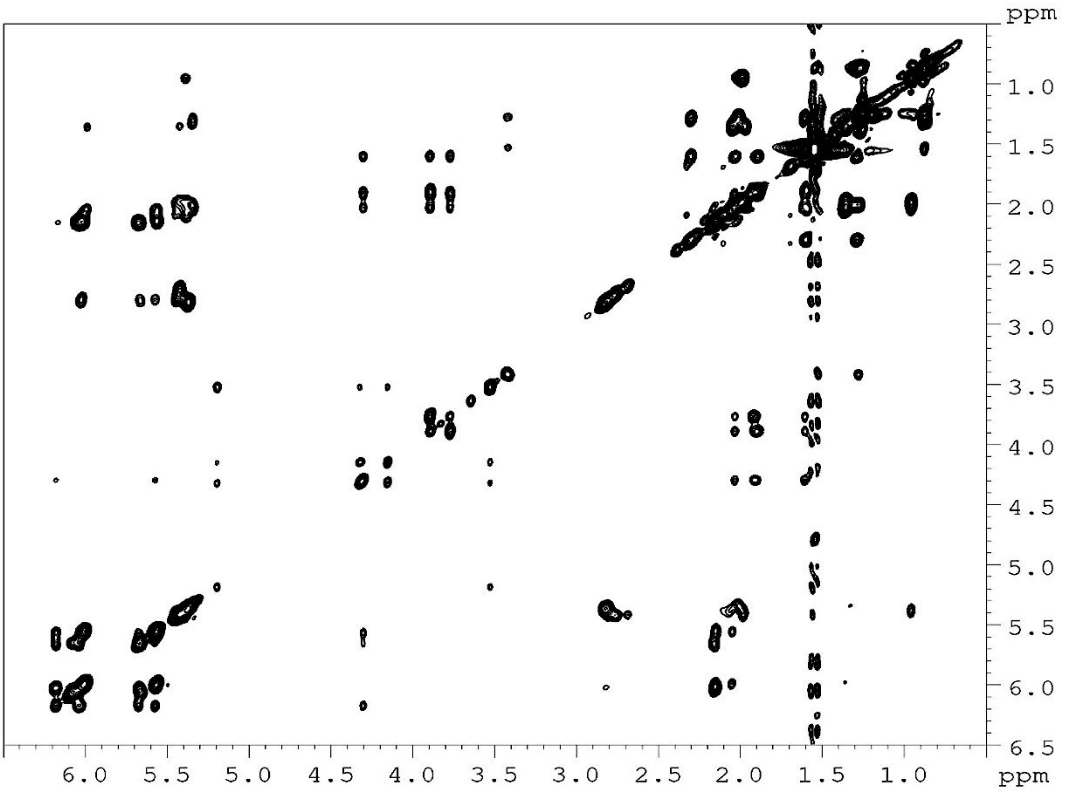

S9. HSQC NMR (600 MHz, $CDCl_3$) of compound **1**.

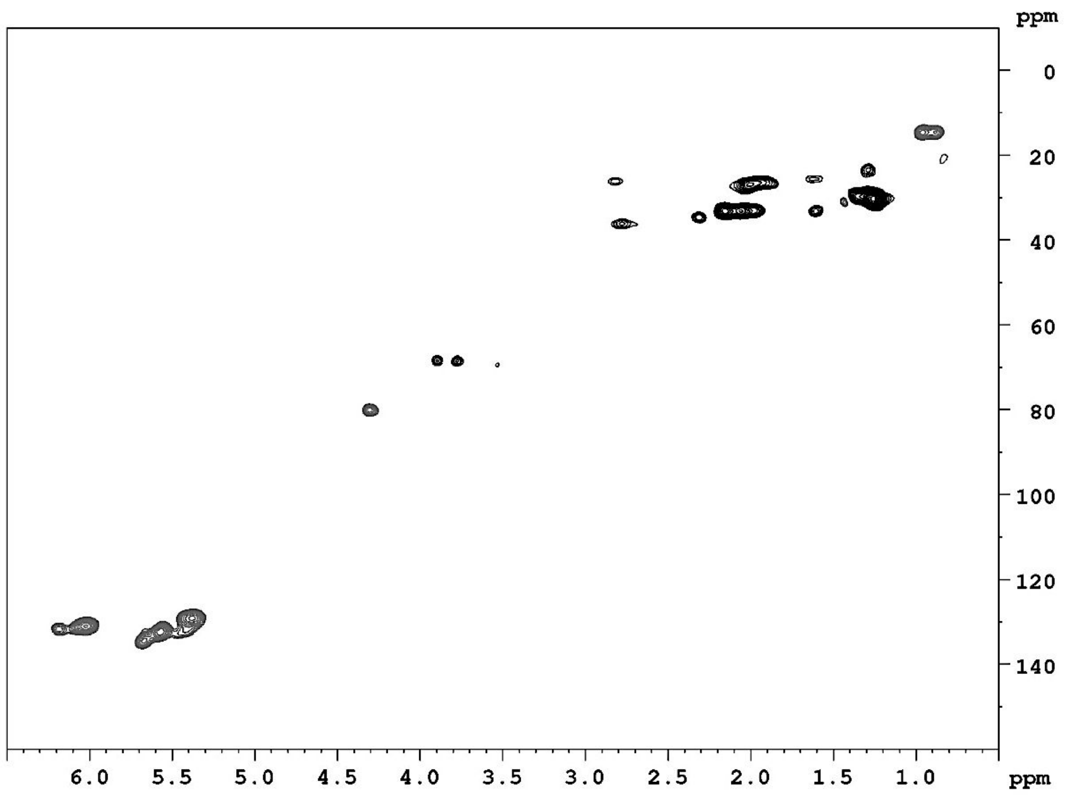

S10. HMBC NMR (600 MHz, $CDCl_3$) of compound **1**.

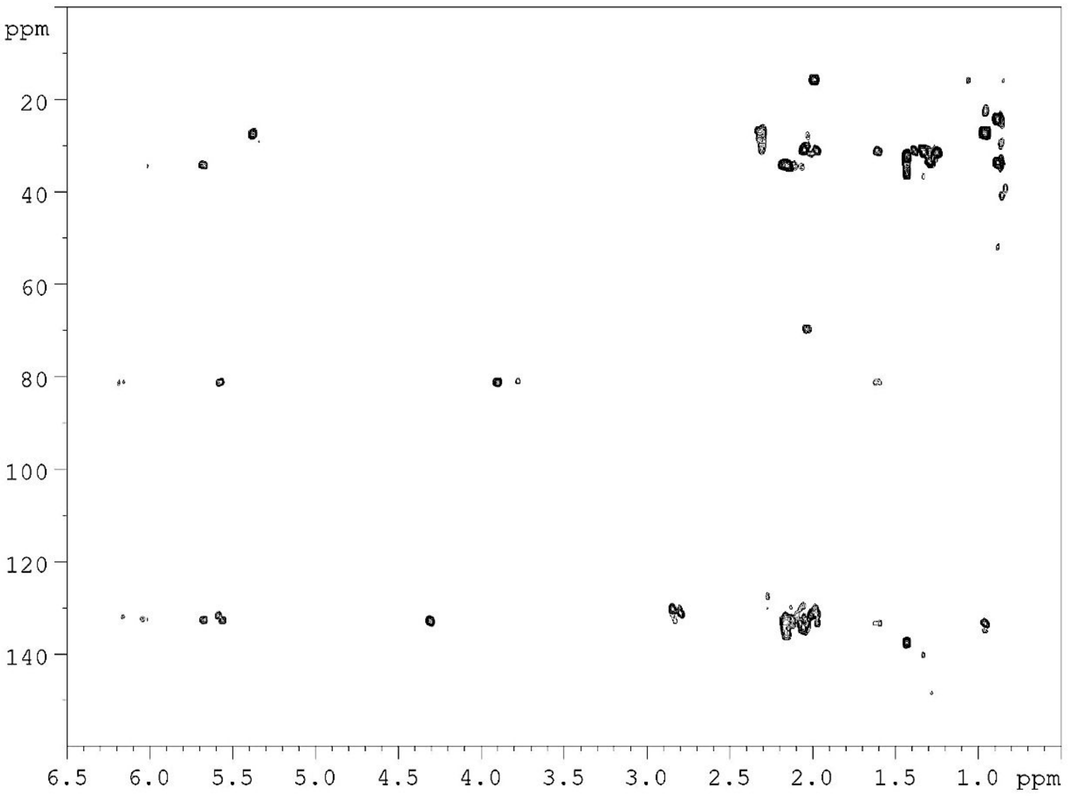

S11. ^{13}C NMR (150 MHz, $CDCl_3$) of compound **1**.

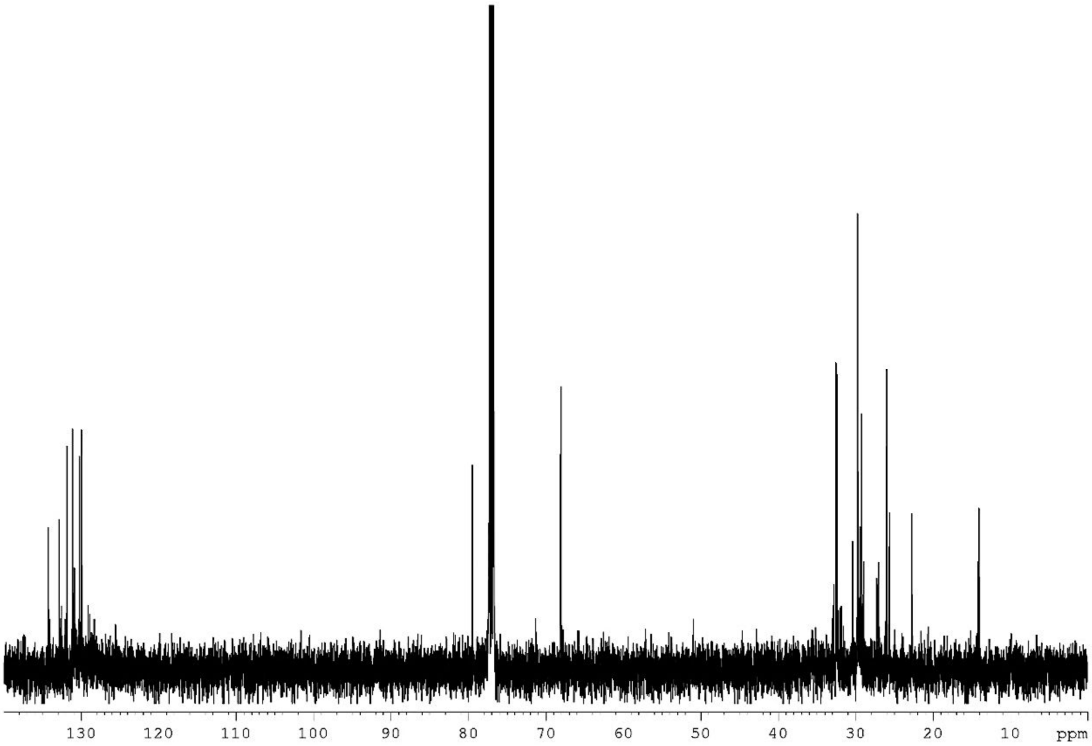

S12. HRESIMS of compound **1.**

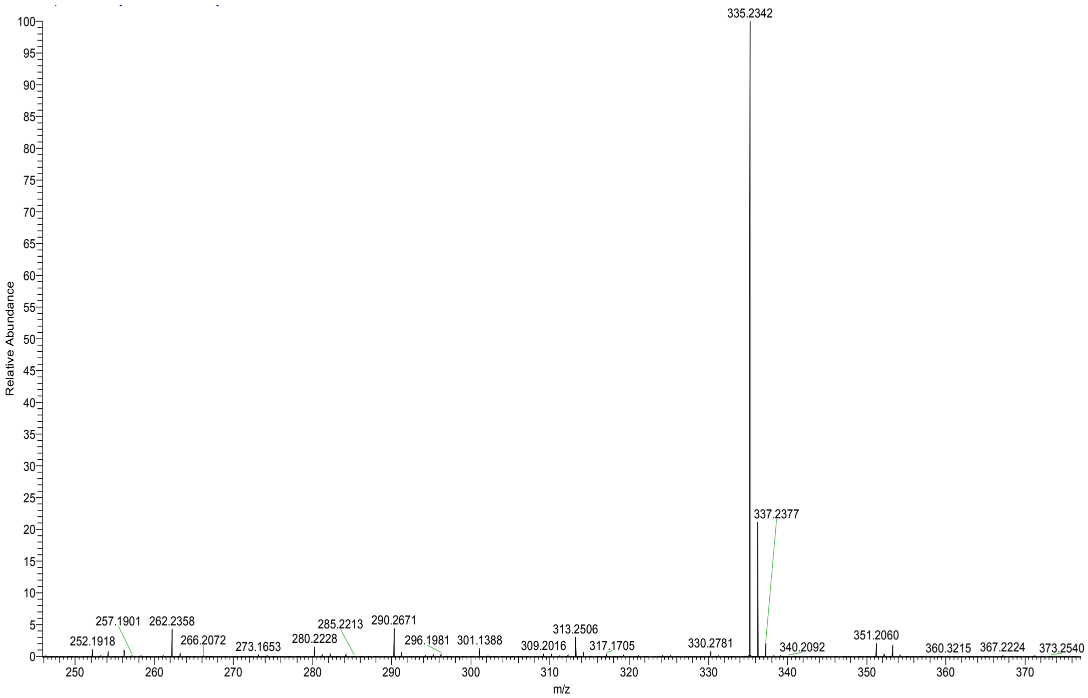

S13. UV of compound **1** (MeOH).

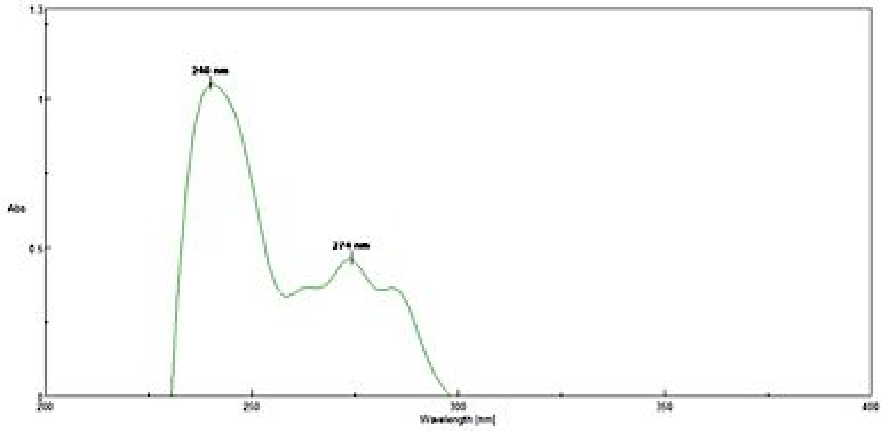

S14. ^{1}H NMR (600 MHz, $CDCl_3$) spectrum of compound **2**.

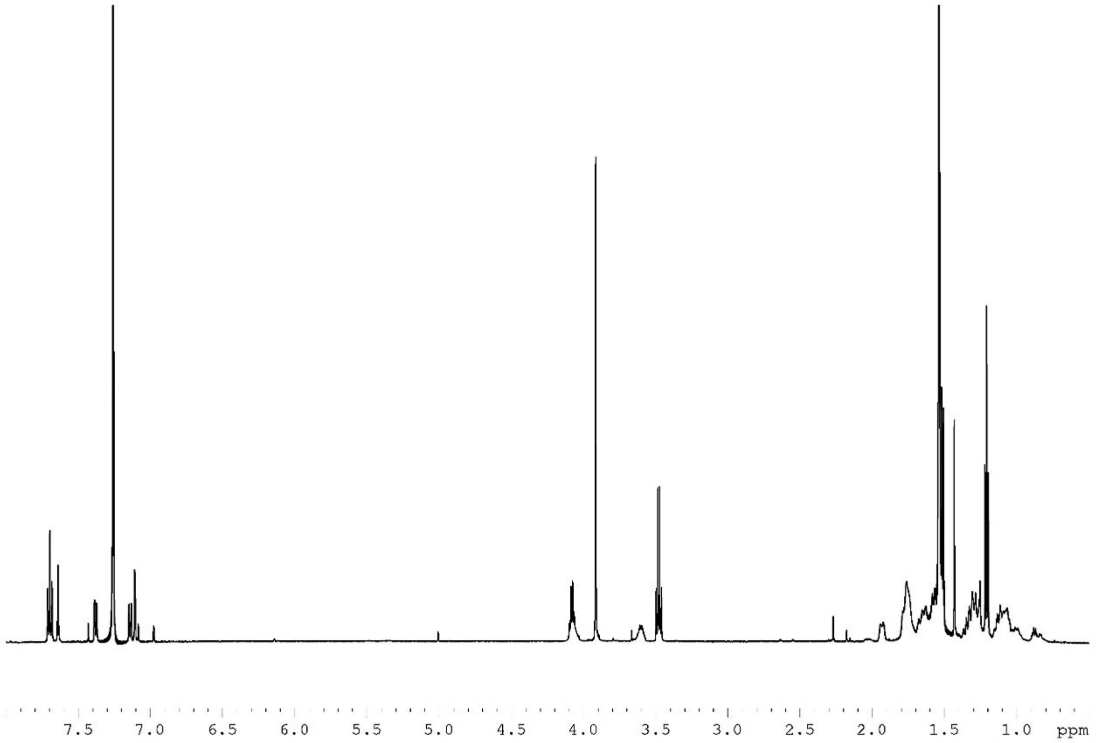

S15. ^{1}H NMR (600 MHz, $CDCl_3$) of compound **3**.

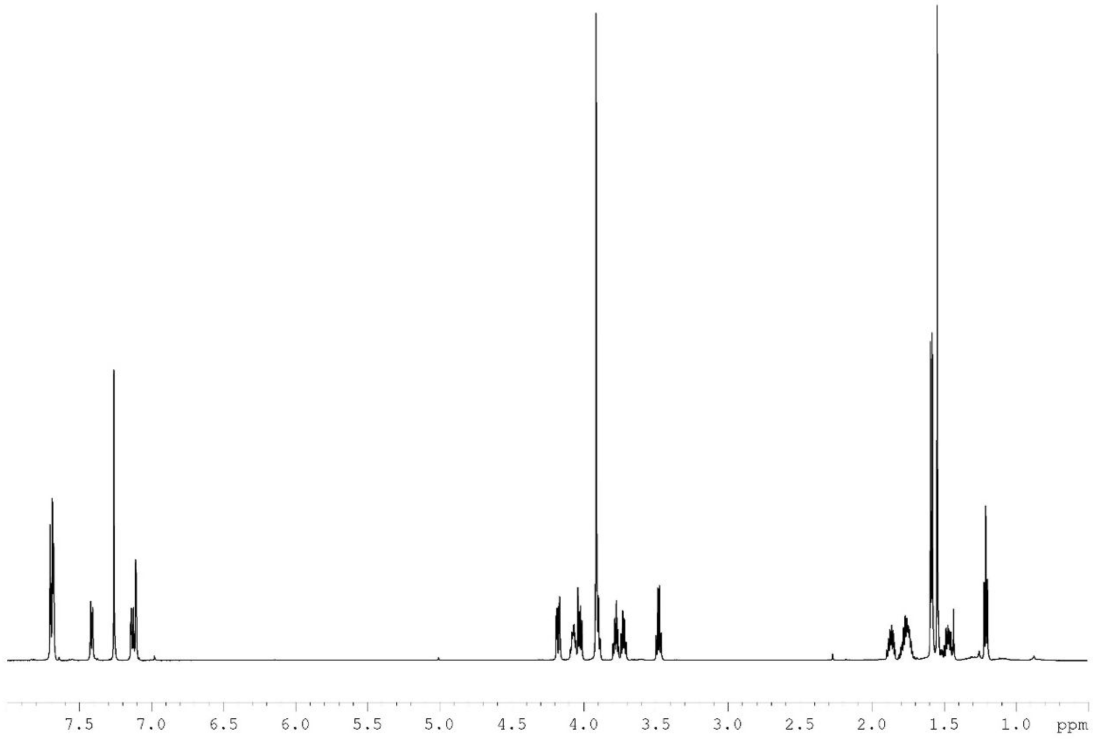

S16. Comparison between ^{1}H NMR (600 MHz, $CDCl_3$) spectra of compounds **2** (RR, green line), **3** (SR, red line) and derivative of **1** (blue line).

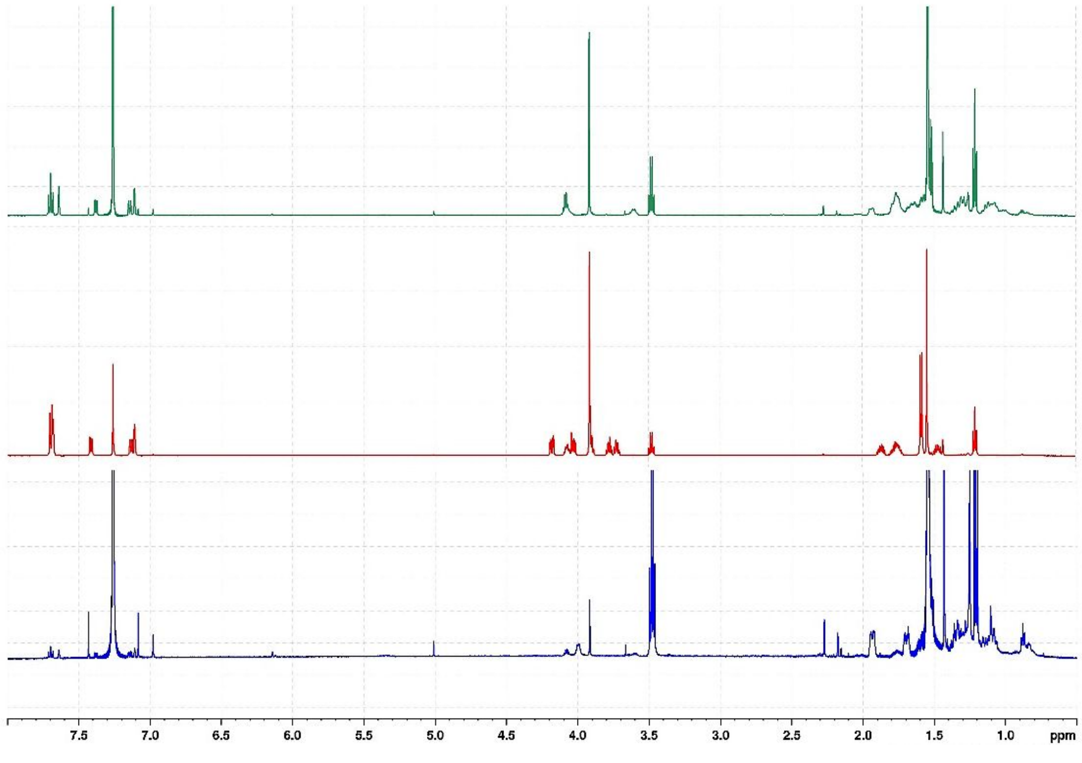

Supplement: Supplementary file 1 [file np5c01409_si_001.pdf]
